# Design and Validation of a Dielectric Method-Based Composite Material Curing Monitoring Platform

**DOI:** 10.3390/s25061686

**Published:** 2025-03-08

**Authors:** Wenfeng Yang, Xinguang Yin, Shaolong Li, Shuaicai Liu, Ran Zhang, Yu Cao, Bowen Yang, Hongshuai Huang

**Affiliations:** 1 College of Aviation Engineering, Civil Aviation Flight University of China (CAFUC), Guanghan 618307, China; zqnr7z_g931lw4zso@163.com (X.Y.); zlishaolong@163.com (S.L.); 13882665957@163.com (B.Y.); click010828@163.com (H.H.); 2 COMAC Composites Center, Shanghai 201324, China; liushuaicai@comac.cc; 3 The Second Research Institute of Civil Aviation Administration of China, Chengdu 610041, China; zhangran@caacsri.com; 4 Intelligent Mfg Inst Laser & Optoelect, Wenzhou University, Wenzhou 325035, China; yucao@wzu.edu.cn

**Keywords:** composites, dielectric analysis, online monitoring, curing monitoring platforms

## Abstract

Monitoring the curing process is crucial for guiding and optimizing the curing procedures of composite material repair patches. Traditional embedded online monitoring methods are limited in their ability to track the curing process of these patches. This paper presents a composite material curing monitoring platform designed using dielectric methods. It integrates temperature control, pressure control, dielectric signal acquisition, control and display modules, and is specifically tailored for bag molding curing of repair patches. The platform measures the ionic viscosity of T300 2019B composites, analyzes the curing index, and correlates it with DSC-cured degree tests. The results indicate that the multiple ionic viscosity curves obtained from monitoring exhibit consistent trends, with correlation coefficients between curves exceeding 0.96. The changes in curing index align with the changes in curing degree, demonstrating that the platform can reliably and accurately monitor the ionic viscosity of repair patches. This platform enables effective monitoring of the ionic viscosity during the curing process of composite material repair patches.

## 1. Introduction

Carbon fiber reinforced plastics (CFRP) are widely used in civilian aircraft structures due to their high strength, rigidity, and lightweight properties [1]. Damage to aircraft composite structures can compromise structural integrity and threaten flight safety, necessitating repair of the damaged areas. In the repair process, the curing of composite patches is a critical step [2]. Variations in the types of damage to aircraft composite structures dictate the dimensions of different composite repair patches, resulting in varying curing times [3,4]. Inadequate curing of composite materials can lead to reduced thermal and mechanical load capacity [5], while over-curing does not significantly enhance the material’s mechanical properties [6,7], but does increase curing time and costs. Therefore, effective online monitoring methods are urgently needed to ensure high-quality and efficient curing of composite repair patches during aircraft structural repairs.

Current online monitoring methods for composite materials include energy conversion techniques [8], optical fiber sensing [9], dielectric sensing [10], carbon nanotube sensing [11], and ultrasonic sensing [12]. These methods rely on principles such as energy conservation, light propagation characteristics, ionic viscosity changes, electrical conductivity of carbon nanomaterials, and ultrasonic wave propagation to monitor the curing process of composite materials. Energy conversion-based curing monitoring methods depend on thermocouples to measure average temperature. For large repair patches, accurate temperature measurement requires multiple thermocouples, leading to complex wiring. Optical fiber sensors require specialized technical skills to operate [13]. Carbon nanotube sensors, primarily composed of carbon nanotubes [14], are costly to produce, increasing the monitoring expense per use. Ultrasonic monitoring relies on probes to emit and receive signals, which can be affected by material thickness during curing [15], and is unsuitable for monitoring curved repair patches. Consequently, these methods are not ideal for curing monitoring of repair patches. To address these issues, it is necessary to develop suitable online monitoring methods that ensure synchronous curing monitoring, while maintaining low costs, high reliability, portability, ease of operation, and insensitivity to the shape and size of the repair patches. Designing a composite material curing monitoring platform suitable for field maintenance processes holds significant application value.

While dielectric methods have been employed by researchers to monitor ionic viscosity and estimate the degree of cure, these studies primarily focus on theoretical aspects rather than practical applications [16,17,18]. This paper addresses the practical need for high-quality and efficient curing of composite repair patches by designing a curing monitoring platform based on Dielectric Analysis (DEA). The platform includes a temperature control module, a pressure control module, a dielectric signal acquisition module, and a control and display module. It is capable of monitoring the ion viscosity during the curing process of the repair patches and obtaining the curing index.

## 2. Design of a Composite Material Curing Monitoring Platform

### 2.1. Platform Design Principles

**Figure 1:**
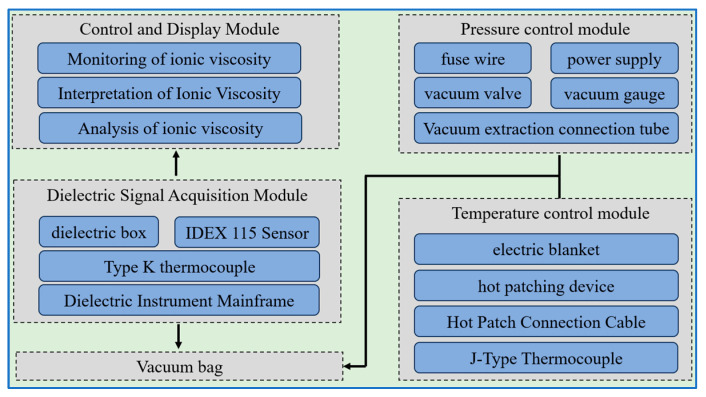
Structure of the curing monitoring platform.

Specialized dielectric sensors are inserted into composite materials. Measurement zones are connected via wires to a dielectric instrument, allowing for the monitoring of ionic viscosity, analysis of the material’s curing state [19,20], and determination of the degree of curing [21,22,23,24]. Curing devices used in conjunction with the dielectric instrument include ovens [25], air ovens [26], and small hot presses [20,23]. Existing dielectric platforms are inadequate for monitoring the curing of repair patches, which are used to address structural damage on aircraft. Consequently, within the framework of aircraft field maintenance, a composite material repair patch curing monitoring platform is designed. This platform is based on practical requirements for monitoring repair patches and ensures interactive connectivity between devices. It comprises four main modules: the temperature control module, pressure control module, dielectric signal acquisition module, and control and display module. The control and display module is responsible for monitoring, analyzing, and interpreting ionic viscosity data. The temperature control module consists of a thermal compensator, electric blanket, and Type J thermocouples, which regulate and control temperature. The dielectric signal acquisition system includes a DEA instrument, dielectric box, dielectric sensors, and Type K thermocouples. This module outputs voltage signals, receives ionic viscosity signals, and transmits the data to the control module. The structure of the dielectric-based composite material curing monitoring platform is shown in Figure 1.

### 2.2. Platform Hardware Design

To fulfill the requirements for patch curing and dielectric monitoring, the platform hardware system was designed based on the principle of “cost-effectiveness”, with the approach of “overall planning and phased implementation”.

#### 2.2.1. Temperature Control Module

The selection of the heating device for the temperature control module depends on the operational environment and the required temperature control accuracy. The HCS9000B thermal compensator (manufactured by HEATCON, USA; weight: 6.8 kg; dimensions: 45.7 cm × 33.02 cm × 14.6 cm) features a curing temperature display of up to 260 °C, operates within an environmental temperature range of 0–50 °C and a humidity range of 0–93%, and maintains a temperature control accuracy of ±1 °C. Its advantages include controllable heating and cooling, ease of operation, compact size, portability, and high temperature control accuracy, making it suitable as the platform heating device.

#### 2.2.2. Pressure Control Module

The pressure control module’s pressure regulation device must be compatible with the thermal compensator model used in the temperature control module. It also needs to integrate a vacuum gauge, fuse, and power switch to ensure effective air extraction and accurate negative pressure measurements. The HCS2055-04 air source electric pump (manufactured by HEATCON, Tukwila, WA, USA; weight: 13 kg; dimensions: 60.96 cm × 38.1 cm × 22.86 cm; air extraction rate: 472 cm^3^/s) provides a vacuum environment for sealed bags. Due to its compact size, convenient box-like packaging for portability, and compatibility with the thermal compensator, it has been selected as the primary pressure control device for the module.

#### 2.2.3. Dielectric Signal Acquisition Module

The dielectric signal acquisition module primarily employs the DEA instrument manufactured by the German compony NETZSCH, with the dielectric sensor serving as its core component. To identify a sensor suitable for the curing monitoring platform while maintaining cost efficiency, a comparative analysis of various sensor models was conducted. The IDEX 115 sensor by NETZSCH, the most economical option, was determined to be suitable. This sensor can tolerate temperatures up to 200 °C and offers a sensing area of 233 mm^2^, comparable to or exceeding that of other sensor models. Preliminary experiments demonstrated that the IDEX 115 sensor is capable of effectively monitoring ionic viscosity. Consequently, the IDEX 115 sensor was selected for integration into the curing monitoring platform.

#### 2.2.4. Control and Display Module

**Figure 2:**
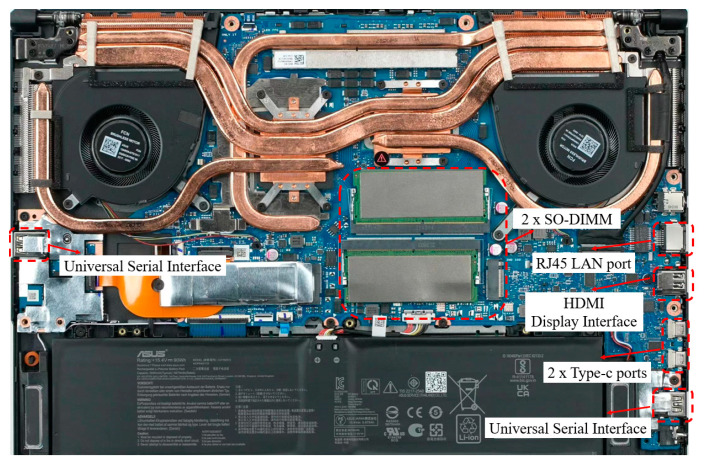
Physical drawing of the control unit.

**Figure 3:**
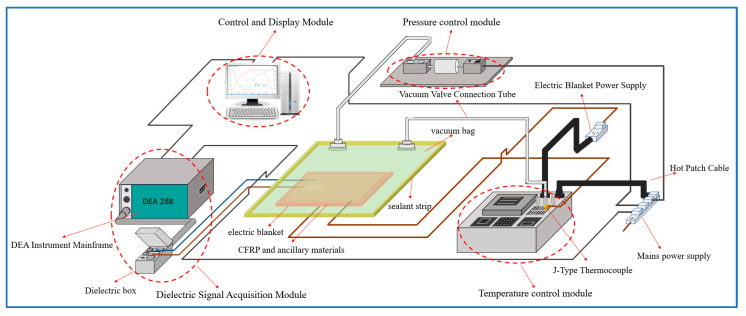
Module application schematic.

In addition to managing system performance, the control module must analyze the ion viscosity data obtained in real-time to assess changes in the composite material’s curing state. Consequently, the control module requires rapid data processing capabilities and high-performance peripheral interfaces to balance real-time data display with ion viscosity analysis. For this purpose, the FX507ZM computer was selected, **it was manufactured by Chinese Asus**, Taiwan, China, featuring a 2.3 GHz Core i7-12700H 14-core processor. The motherboard includes an RJ45 LAN port, two serial ports, two Type-C ports, and an HDMI display port to meet the computational needs for data interaction. The display module chosen is a 15.6-inch ASUS IPS screen with WQHD resolution. The physical control unit is shown in Figure 2, while schematic diagrams of the modules are depicted in Figure 3.

### 2.3. Platform Software Construction

The dielectric real-time monitoring software “NETZSCH DEA Measurement” (version DEA 288) and the dielectric analysis software “Proteus^®^ Analysis” (version 7.1) were installed on the computer. These work in conjunction with the Data Capture and Analysis Software, which was provided with the thermal repair instrument. This integrated system enables the curing and monitoring of repair patches.

**Figure 4:**
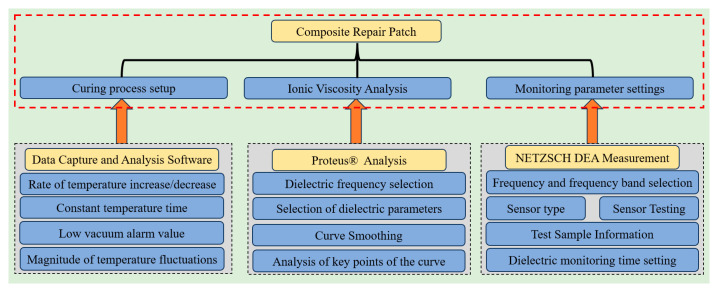
Platform software architecture.

Among them, the dielectric real-time monitoring software NETZSCH DEA Measurement primarily enables the monitoring of ionic viscosity during the curing process of composite materials. Before initiating monitoring, it was essential to configure the test frequency and monitoring duration. Additionally, the sensor was tested in air to ensure it was functioning correctly. This involved observing the data for voltage amplitude, current amplitude, and phase angle, and comparing them to the manufacturer’s manual to confirm normal operation. The dielectric analysis software Proteus^®^ Analysis was used to process and analyze the ionic viscosity curves, including tasks such as curve smoothing and the identification of critical points. The Data Capture and Analysis Software, which came with the hot patch instrument, controlled and monitored the temperature inside the vacuum bag. Together, these software applications facilitated the curing and monitoring of composite patches. The platform’s software architecture is illustrated in Figure 4.

## 3. Composite Curing Monitoring Platform Integration and Application

### 3.1. Integration of Curing Monitoring Platforms

**Figure 5:**
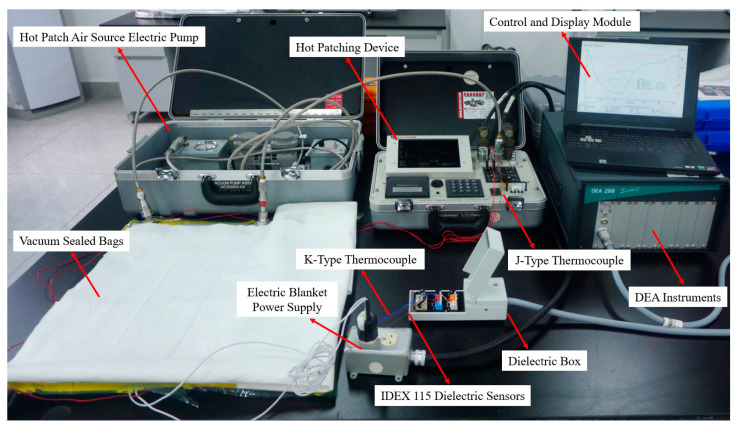
Composite curing monitoring platform.

Properly positioning the temperature control module, pressure control module, dielectric signal acquisition module, and control and display module is essential. The pressure control module and the dielectric signal acquisition module should be placed separately to prevent vibrations from the pneumatic pump from affecting the DEA main unit and dielectric box, which could impact the measurement of ion viscosity. Additionally, K-type and J-type thermocouples should be inserted into the sealing bags from different directions to avoid short-circuiting due to contact, which could affect temperature measurement. Each module’s power supply was equipped with a protective circuit to ensure stable platform operation. The composite material curing monitoring platform is illustrated in Figure 5.

### 3.2. Sample Preparation

The CFRP material used in this study was T300 2019B carbon fiber/epoxy composite, produced by Weihai Lanke Composite Materials Co., Ltd. (Weihai, China). During the experiment, two layers of 15 cm × 10 cm prepreg were laid to simulate the curing of aircraft composite repair patches. To address the potential short-circuiting of the dielectric sensor electrodes due to contact with carbon fiber, glass fiber cloth was used as an insulating layer to separate the dielectric sensor from the composite material.

### 3.3. Platform Parameters Configuration

The parameters for editing the composite material curing process and ion viscosity signal testing are as follows: (1) vacuum should be applied until the pressure is less than 75 kPa (22 inches of mercury); (2) heating phase: from 25 °C to 125 °C at a heating rate of 1 °C/min; (3) soaking phase: maintain 125 °C for 120 min; (4) cooling phase: from 125 °C to 40 °C. Given that ion viscosity signals are influenced by the frequency of the alternating electric field, and based on preliminary experiments indicating better results within the 1–10 Hz range, this study selects ion viscosity curves obtained at a frequency of 10 Hz for analysis.

### 3.4. Experimental Methods

#### 3.4.1. Platform Reproducibility Testing

To validate the stability of ion viscosity monitoring on the platform, reproducibility experiments were conducted. A total of seven ion viscosity measurements were performed, and the reproducibility of the curves was analyzed from three perspectives: overall trend, mean and standard deviation, and correlation coefficient.

#### 3.4.2. Determination of the Curing Index

Considering that the curing reaction of composite materials primarily occurs during the period when the viscosity of the matrix resin increases from its minimum value, the curing index is determined by calculating the ion viscosity data from the lowest viscosity point to the end of the isothermal stage using Formula (1) [27].

(1) $$\alpha_{t}=\frac{\log(\rho_{t})-\log(\rho_{\min})}{\log(\rho_{\max})-\log(\rho_{\min})}$$

In this context, $\log(\rho_{t})$ represents the decimal logarithm of the ion viscosity signal measured at time *t*, $\log(\rho_{\min})$ corresponding to the minimum ion viscosity. The maximum ion viscosity, denoted as $\log(\rho_{\max})$, is determined by the peak of the ion viscosity curve. The curing index is represented as $\alpha_{t}$.

#### 3.4.3. Curing Degree Acquisition

Isothermal differential scanning calorimetry (DSC) tests were conducted using a **DSC 214 instrument manufactured by Netzsch**, **Germany**. Composite materials weighing between 5 and 10 mg were measured and subjected to the curing processes employed by the platform, during which the thermal flow variations were recorded. Subsequent non-isothermal DSC tests involved heating at a rate of 10 °C/min up to 150 °C to assess the residual heat of the materials. The degree of cure of the materials was determined from the isothermal DSC tests and calculated using Equation (2) [28].

(2) $$\alpha (T)=1-\frac{\Delta H_{p}}{\Delta H_{total}}$$

The total area under the exothermic peak represents the material’s total heat release, $\Delta H_{total}$; $\Delta H_{p}$ denotes the area under the exothermic peak from time zero to the actual time *t*; α(T) signifies the degree of cure of the material.

## 4. Results and Discussion

### 4.1. Analysis of the Platform’s Monitoring Reproducibility

**Figure 6:**
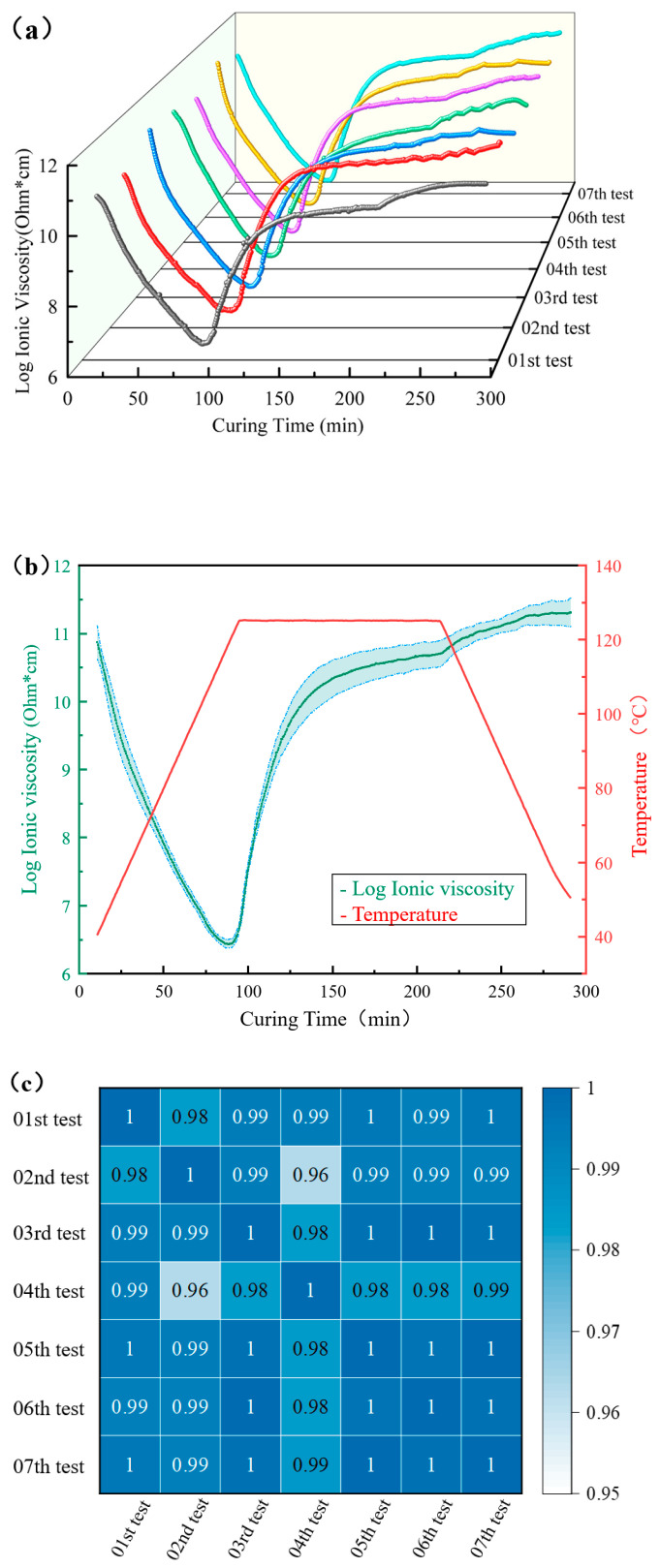
Results of ionic viscosity curve processing ((**a**) 3D waterfall plot; (**b**) error band plot; (**c**) thermogram).

To minimize the impacts of environmental humidity, uneven resin distribution in the patch substrate, and voltage stability on ion viscosity monitoring, this platform conducted seven ion viscosity experiments. The resulting ion viscosity curves, as depicted in Figure 6a, exhibited consistent trends across all trials. In the initial stages of curing, the matrix resin is in a near-solid state, resulting in high ionic resistivity and, consequently, high ionic viscosity. As the temperature increased, the resin gradually softened and became more fluid, enabling the internal molecules and ions to gain increased kinetic energy and mobility, which correspondingly decreased the ionic viscosity.

Upon reaching a certain temperature, the resin enters the initial curing phase, initiating polymerization and cross-linking reactions. As a result, the average molecular weight within the resin gradually increases. The enlargement of molecules and their subsequent entanglement reduce the mobility of charged ions between these larger molecules, leading to increased ionic resistivity. Initially, the rate of the curing reaction is relatively low, and the decrease in ionic viscosity due to the temperature rise is predominant, as evidenced by the continuing decline of the ionic viscosity curve, albeit at a reduced slope. This decline reaches a nadir, at which point the reduction in ionic viscosity caused by the temperature rise and the increase caused by cross-linking reach a rate equilibrium. Subsequently, the rate of the cross-linking curing reaction continuously increases and becomes the dominant factor, manifesting as an accelerated increase in ionic viscosity [20].

Upon entering the isothermal phase, the resin continues to undergo cross-linking and curing, gradually forming a supportive three-dimensional scaffold structure internally. This leads to an increase in the resin’s viscosity, constraining the spatial mobility of ions, and successively reducing their mean free path, which is reflected in a rapid increase in ionic viscosity. In the later stages of the isothermal phase, the resin essentially completes the formation of the supportive three-dimensional scaffold structure, and the increase in ionic viscosity slows, indicating that the curing of the patch matrix resin is nearly complete. Following this, the cooling phase begins. During this stage, the impact of cross-linking and curing on ionic viscosity diminishes, and the primary influence is the resin’s negative temperature coefficient of resistivity [29]. As the temperature decreases, the thermal motion of ions within the resin eases, slowing their migration speed through conductive channels, which manifests as an increase in ionic viscosity. The monitored ionic viscosity of the composite material patches during curing can reflect the actual curing status of the patch.

To visually demonstrate the differences among the seven ionic viscosity curves, the mean and standard deviation of the values from these curves were calculated and used to create an error band graph, as shown in Figure 6b. During the heating phase, the band of error gradually decreases. This reduction occurs because, as the temperature increases, the matrix resin softens and its viscosity decreases. Additionally, since the same material was tested seven times, the differences in the values of the seven ion viscosity curves also diminish. In the isothermal phase, as the matrix resin undergoes cross-linking and curing, the error band width gradually increases, although the maximum bandwidth remains below 0.3. The consistency in the trend of the ionic viscosity curves during this phase, all showing a slow increasing trend, indicates the stability of the monitoring platform’s multiple curves. When the temperature decreases, the error width remains largely constant, further indicating that the material has completed its curing process.

To further illustrate the repeatability of ionic viscosity measurements monitored by the platform, a heatmap, as shown in Figure 6c, was introduced to visualize the correlation among the seven ionic viscosity curves. The results indicate that the correlation coefficients between these curves range from 0.96 to 1, demonstrating a high degree of correlation among them. Therefore, this curing monitoring platform can reliably monitor the ionic viscosity throughout the curing process of the patch.

### 4.2. Access and Validation Platform Curing Index

**Figure 7:**
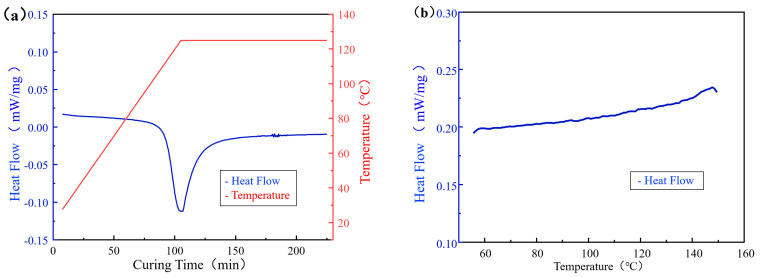
DSC experimental curves ((**a**) isothermal DSC experimental curves; (**b**) non-isothermal DSC experimental curves).

**Figure 8:**
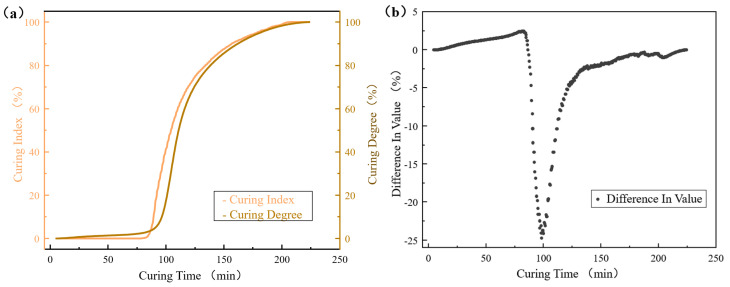
Material curing ((**a**) curing index, curing degree curve; (**b**) difference between curing degree and curing index).

The heat flow curves for the composite materials during the isothermal and non-isothermal DSC tests are illustrated in Figure 7. A prominent exothermic peak occurs during the isothermal curing process, with no corresponding peak in the subsequent non-isothermal process, indicating that the material’s curing is complete in the isothermal phase. The degree of cure was determined by integrating the area under the exothermic peak using Equation (2), as shown in the curing degree curve in Figure 8a. The value of the material’s degree of cure increases progressively with the continuation of the isothermal curing.

Using Equation (1), one ionic viscosity curve was selected from seven for analysis to obtain the curing index, as illustrated by the curing index curve in Figure 8a. Over time, the curing index of the composite material progressively increased until it reached 100%.

The difference between the curing degree and the curing index was calculated, and the corresponding scatter plot is shown in Figure 8b. The results indicate that the difference is less than 5% for curing times ranging from 0 to 90 min and from 120 to 220 min. However, the difference exceeds 5% during the 90–120 min period, which may be attributed to the influence of the composite material’s thermal conductivity, thickness, and specific heat on the curing reaction rate [30]. Additionally, the size and weight of the patches used in the curing monitoring platform are significantly larger than those of the specimens tested by DSC, and this disparity may affect the curing reaction rate due to the interaction of the aforementioned factors. Despite this, the phase only represents 13.64% of the total duration, and the overall trends in both the curing index and the degree of cure are similar. Therefore, the curing in-dices measured by the platform closely correspond to the actual changes in the material’s degree of cure.

To further illustrate the accuracy of the monitoring platform, the correlation between the curing index and the curing degree was quantified by calculating the Pearson correlation coefficient, which was found to be 0.991. This indicates a highly significant positive correlation between the two variables. Consequently, the curing index can be used to predict changes in the actual curing degree, confirming that this monitoring platform can accurately track the ionic viscosity during the curing process of the repair patches.

## 5. Conclusions

This paper presents a monitoring platform designed for curing composite material patches, which utilizes the platform to monitor the ionic viscosity and obtain the curing index of the composite patches. Additionally, the degree of cure was assessed using differential scanning calorimetry (DSC). The findings are as follows:

(1) The platform demonstrates high repeatability in monitoring the ionic viscosity of the same patch during the curing process. This stability suggests its applicability in consistently monitoring the curing of composite material patches in aircraft structural repairs.
(2) The curing index obtained from the platform closely aligns with the actual changes in the degree of cure of the patch, indicating that the measured curing index can effectively represent the actual degree of cure. The platform accurately monitors the ionic viscosity throughout the curing process.

Consequently, this curing monitoring platform can stably and accurately monitor the ionic viscosity during the patch curing process, proving suitable for curing monitoring in the repair of composite patches, and holds significant potential for broader application.

## Data Availability

The data presented in this study are available on request from the corresponding author.
